# Effects of a Social–Emotional Learning Intervention on Social–Emotional Competencies and Behavioral Problems in Elementary Students Amid COVID-19

**DOI:** 10.3390/ijerph21091223

**Published:** 2024-09-17

**Authors:** Raquel Raimundo, Sofia Oliveira, Magda Sofia Roberto, Alexandra Marques-Pinto

**Affiliations:** 1 Gulbenkian Knowledge Academies, Calouste Gulbenkian Foundation, 1067-001 Lisbon, Portugal; 2 ISCTE—Instituto Universitário de Lisboa, Business Research Unit (BRU-Iscte), 1649-026 Lisbon, Portugal; sofia.oliveira@iscte-iul.pt; 3 CICPSI— Centro de Investigação em Ciência Psicológica, Faculdade de Psicologia, Universidade de Lisboa, 1649-013 Lisboa, Portugal; msroberto@psicologia.ulisboa.pt (M.S.R.); ampinto@psicologia.ulisboa.pt (A.M.-P.)

**Keywords:** baseline levels, gender, intervention gains, social–emotional learning, socioeconomic status

## Abstract

This study investigated whether a social–emotional learning program, implemented over a one-year period, could lead to gains in social–emotional competencies and to a reduction in internalizing and externalizing problems in the context of the COVID-19 pandemic. Furthermore, the program analyzed how students (boys vs. girls) with varying levels of social–emotional competencies and externalizing and internalizing problems, and from different socioeconomic backgrounds, were differently affected. The program was applied to 358 Portuguese third- and fourth-grade students (51.4% boys, *M*_age_ = 8.56; *SD* = 0.82). Self-report (students) and hetero-report (teachers) questionnaires were administered before and after the intervention. Linear mixed-effects models were computed to test intervention impacts. Significant intervention gains were noted in social–emotional learning competencies, namely emotional knowledge, social competence, peer relations, self-management, and academic behavior, and in externalizing (social problems) and internalizing (anxiety) problems. No effects were found in aggressiveness. Students with lower social–emotional competencies and higher externalizing and internalizing problems at baseline profited more from the program. Gender moderated both emotional knowledge and social problems, and socioeconomic status only moderated social problems. Findings highlight the effectiveness of this social–emotional learning program, especially for students facing initial challenges. Recommendations for future research, acknowledging limitations and strengths, are discussed.

## 1. Introduction

### 1.1. The Impact of the COVID-19 Pandemic on Youth Mental Health and Social Inequality

Over the last decade, education and mental health have been cited as social, political, and scientific priority issues requiring attention [1].

The COVID-19 pandemic has represented a serious threat to mental health, particularly among young people [2]. However, a positive benefit of the COVID-19 pandemic is that it has turned the spotlight on youth mental health [3] by revealing increased rates of depression, tension, and anxiety among young people during the COVID-19 crisis [4].

Although mental health among young people had already been identified as a problem prior to the pandemic [5], the available data show that since the outbreak of COVID-19, young people’s mental health has been disproportionally affected in comparison with that of other age groups [2]. According to a study of 80,000 children and young people under the age of 18 years, the rates of clinically significant depression and anxiety have doubled, with one in four young people experiencing depression, one in five experiencing anxiety, and children presenting higher anxiety rates [4].

The disruption to life activities, such as the shift from face-to-face to online learning, the restrictions on leisure and sports activities, and the partial or total interruption of social participation has seriously undermined interpersonal relations, leading to a loss of social relations and causing an increase in depression, anxiety, feelings of isolation and loneliness, somatic complaints, and aggressive behavior and a decrease in psychological strength, autonomy, and overall psychological well-being [6].

Moreover, the pandemic has hit certain groups of young people harder than others [7], affecting their mental health unequally [3]. Indeed, the COVID-19 pandemic has had a tremendous impact on young people’s lives, especially the most vulnerable [3], likely prolonging their disadvantages in school [8]. Young people with pre-existing mental health issues found it more difficult to access support services during this period and suffered the psychological consequences of the pandemic to a greater extent [2,9]. Children and adolescents who faced higher risks of psychological health problems were those with families in vulnerable situations, previous psychological health problems, or adverse childhood experiences [4].

Furthermore, the crisis has heightened existing economic and social inequalities [3]. Although all children were at risk of falling behind, young people from lower-income backgrounds were more vulnerable to social exclusion and related mental and emotional consequences [7], including more daily stressors that may interfere with normal, healthy stress responses [10].

### 1.2. The School’s Role in Promoting Social–Emotional Learning and Well-Being

Growing awareness of the increasing number of school-aged children and youth presenting with behavioral, emotional, and mental health problems, with relevant impacts on outcomes as diverse as academic performance, obesity, and risk behaviors [11], has led to an important and consensual change in perspective on the role of the school, which should encompass not only the instruction of academic content and skills [12] but also the provision of support for children’s emotional education and development [13].

In fact, in addition to their role in academic learning and performance, schools are now expected to actively participate in the promotion of lifelong success, mental health, and well-being of young people [14,15,16]. This is often accomplished through the implementation of universal social–emotional learning (SEL) programs, which aim to improve learning, promote emotional well-being, and prevent problem behaviors through the development of social and emotional competencies [13,17]. These competencies are thought to facilitate students’ academic performance, positive social behaviors, and social relationships during the academic years; to reduce behavioral problems and psychological distress; and help to prepare young people to succeed in college, work, family, and society [18,19].

Nowadays, SEL is defined as the process through which children and adults acquire and effectively apply the knowledge, attitudes, and skills needed to manage their emotions, set and achieve positive goals, feel and show empathy for others, establish and maintain positive relationships, and make responsible decisions [20]. More specifically, Weissberg et al. [21] have identified a set of core social and emotional competencies (self- and social awareness, self-management, relationship skills, and responsible decision making). These five key-competencies are common to all consortia for/approaches to SEL [12,22]. Although SEL has a practice-centered origin, two main theoretical frameworks significantly influence its conceptualization and operationalization: (1) the emotional intelligence theory [23], which guides the development of emotionally related areas, and (2) the social skills training movement, grounded in Bandura’s social learning theory [24], which shapes the development of self-regulation and interpersonal relationship areas [25,26].

Research has shown that when effectively implemented, evidence-based SEL programs lead to measurable and potentially long-lasting improvements in various domains of a child’s life [27,28]. Comparing the findings of four large-scale meta-analyses of SEL programs [13,29,30,31] containing outcome data from 356 research reports summarizing the short- and long-term effects of hundreds of thousands of universal school-based SEL programs revealed that the four meta-analyses reached the same general conclusions. SEL programs produced positive benefits in behavioral, attitudinal, emotional, and academic domains for participating students that were evident both immediately after the intervention had ended and in various follow-up periods, depending on the specific outcome in question [12]. More broadly, they contributed to harmonious relationships, to social cohesion and inclusion in communities, to positive attitudes toward individual and cultural diversity, and to equity and social justice [32].

Empirical findings on SEL suggest that successful programs must be evidence-based and both well designed and well conducted [29], i.e., be developmentally and culturally appropriate; promote the generalization of newly-learned skills [11,22,29,33]; include a written manual specifying the conceptual model and the intervention procedures of the program [34]; and adhere to SAFE practices, i.e., ensuring that the program uses a coordinated sequence of activities, promotes active forms of learning, devotes sufficient time and attention to social–emotional skill development, and has clear and specific SEL objectives [29]. Successful SEL programs also have a high level of structure and consistency in program delivery and are carefully monitored to ensure that they are delivered as intended by their developers [35].

Implementation is also likely to be more effective and with longer-lasting outcomes if ongoing training and consultation are provided [36]. Also, regular opportunities for staff to develop these skills must be fostered through high-quality professional development [37] grounded in the most recent advances in the science and practice of systemic SEL [38] to support teachers’ social and emotional development [39].

### 1.3. The “Slowly but Steadily” SEL Program: Development and Impacts

“Slowly but Steadily” [40] is a universal program developed in Portugal for the promotion of social–emotional competencies in a school context. Based on empirical evidence regarding its effectiveness, the “Slowly but Steadily” program was adopted by the Gulbenkian Knowledge Academies as a benchmark methodology for promoting social and emotional competencies in elementary school children. It is also one of three Portuguese programs promoting socio-emotional competencies mentioned in the European Union report on evidence in socio-emotional education [32].

“Slowly but Steadily” is supported by the theoretical reference framework of SEL [22] by integrating competence promotion and youth development frameworks [29] and is based on the best evidence on “what works”. It is based on the theoretical model of emotional competence [41], the ABCD developmental model (affective, behavioral, cognitive, and dynamic) [42], the bio-ecological model [43], and the social learning model [44]. The underlying assumptions are that emotions are important in young people’s lives, not having the competencies to understand and manage them can be disruptive to social and cognitive development [45], and that social–emotional competencies can be learned and taught in schools and require explicit instruction, much like academic skills [33].

“Slowly but Steadily” is a comprehensive program that emphasizes not only the teaching of skills but also how to apply them to meaningful real-life situations to facilitate their generalization. It is implemented in a classroom context in a group-class setting and inserted in the school curriculum. It consists of 21 developmentally appropriate 45–60 min sessions, delivered weekly over an academic year by school psychologists in the teachers’ presence, following a sequenced set of activities that emphasize learning by doing and by interactive and reflexive experiences [40].

The program manual contains session plans, specifying the SEL learning objectives, techniques and strategies to be implemented, materials to be used, and the description of the activities for each session [40]. The techniques and strategies used include didactic instruction, posters, storytelling activities, reflection/brainstorming underlying the most efficient strategies with open-ended questioning, modeling, role playing, constructive feedback, social- and self-reinforcement, and group games. Sometimes, group games are used without prior explicit mention of the concepts to better engage and prepare the children to integrate skill concepts. Reflection/brainstorming, role playing, constructive feedback, and social- and self-reinforcement follow, giving children a chance to practice the skill, the trainer to monitor the level of understanding and skill attained, and the teachers to learn “on the job” how to develop social–emotional competencies and to generalize them to other classes, thus enhancing their impact in the school microsystem [43]. Although teachers are encouraged to take part in the sessions, their level of involvement is often variable. Finally, teachers are encouraged to promote the generalization of competencies during the week by reminding pupils throughout the day to use them (especially before recess). The curriculum is divided into five units, each containing developmentally sequenced sessions to integrate and build on previous learning and offer children a global vision of social–emotional competencies [46].

The first unit focuses on the self-awareness, understanding, and communication of emotions and is divided into four sessions with six activities. One example is the “Emotions Game”, in which children must mimic emotions for their team using cards. The team guesses the emotions, starting with the basic ones and moving on to the more complex emotions. The second unit is dedicated to social awareness, perspective taking, and empathy and has three sessions with four activities. “Being Different” is one of those activities and consists of reading testimonies of children describing a family member or friend with some kind of disability, focusing on the similarities and differences and how one feels when being discriminated against. The third unit relates to emotion regulation, and it is divided in four sessions with three activities. One of them is called “Let’s Overcome Bad Feelings”, in which children are encouraged to describe situations and to describe how their body reacted, how they felt, what they thought, what they did, and what the consequences of their actions were, followed by a brainstorming session in which the most efficient strategies on how to deal with certain emotions are underlined. The fourth unit focuses on interpersonal skills, peer communication (assertiveness), and managing conflicts and has four sessions with six activities. Group games, as well as role playing, in which children must work together as a team are the focus of this unit, including time to reflect on communication skills and how to negotiate in a conflict situation. The last unit is dedicated to responsible decision-making and problem-solving skills and has only one session with two activities, and it is also approached during the previous units. The steps to make a responsible decision are exemplified through stories, and children are encouraged to work in groups and apply what they have learned in other hypothetical situations [40,46].

The results of the quasi-experimental longitudinal empirical studies showed the effectiveness of the implementation of the “Slowly but Steadily” program over one academic year in promoting social and emotional skills and academic performance, as well as reducing externalizing behavior problems in children belonging to the intervention group, compared to the control group. Thus, generalized gains with the program were observed in peer relations and social competence; boys in the intervention group also benefited in self-control and aggression, and the children in the intervention group who had average levels of self-control and peer relations prior to implementation displayed significant improvements in these skills compared to the children in the control group. The gains obtained in these skills with the implementation of the program were independent of the children’s socioeconomic status (SES) [46]. The positive impact of the program was also felt in the medium term, with the children in the intervention group showing gains in academic performance 10 months after implementation compared to the children in the control group. The program was not, however, effective in reducing internalizing behavior problems, and no medium-term gains (“sleeper effects”) were recorded for emotional cognition and anxiety in the follow-up study [47].

Despite the considerable empirical evidence supporting the positive impact of the “Slowly but Steadily” program and suggesting the student characteristics that lead them to benefit differently from it, this is an issue that continues to be controversial in the literature in the area. In fact, considering that various sociocultural factors such as gender, age, SES, and ethnicity may influence children’s social and emotional competencies [48], examining the effects of these moderating variables is crucial for several reasons.

First, adapting SEL programs to account for differences between groups of children may significantly enhance their efficacy and positive effects. For example, gender differences in social and emotional competencies can lead to adjustment challenges, making it essential to tailor SEL interventions accordingly [49]. As Bandura’s social learning theory [44] posits, these differences are shaped by sociocultural norms that children internalize by observing and imitating gender-specific behaviors in their role models. Although the literature acknowledges that both boys and girls demonstrate social and emotional competence in various ways, research suggests that girls tend to develop social competence earlier, while boys are more likely to exhibit aggressive and externalizing behaviors influenced by societal expectations [49,50]. Although SEL programs hold promise, further research is needed to clarify the complex relationships between gender, socioemotional variables, and externalizing behaviors [49].

Second, ensuring that SEL programs are equally beneficial across different population subgroups is vital for the successful scaling of evidence-based interventions [48]. School-based universal SEL programs have the potential either to reinforce existing social inequalities by perpetuating dominant norms or to promote equity by fostering skills that mitigate the effects of such inequalities and create inclusive environments [51]. Although interventions are generally considered most effective for children from less supportive developmental environments, often including those from low-income households [52], research on the impact of SES on SEL interventions has produced mixed results. The results of Durlak et al.’s [29] meta-analysis suggested that school-based social–emotional competence interventions were suitable and effective for all students, and in some cases, these interventions were more favorable for students from low-income families. In Taylor et al.’s [31] meta-analysis, significant positive effects for SEL program participants were found at follow-up across all demographic subgroups, including those determined by SES and ethnicity. In this study, age was significantly negatively related to follow-up effects when examined as an individual predictor, but this finding should be interpretated with caution, since interventions targeting younger children were also delivered over a longer period. Taken together, the research suggests that our understanding of the moderating variables that influence the effects of the program is still obscure [29,31,53], with studies showing contradictory results for the variables of gender, age, SES, and baseline level of externalizing and internalizing problems [47].

### 1.4. The Present Study

Considering the constraints and impacts of the COVID-19 pandemic on students’ previously underlined social and emotional competencies, well-being, and academic performance [3,8], there was an urgent need to intervene to mitigate these impacts on elementary school students.

As the “Slowly but Steadily” program had previously proven to be effective in promoting elementary school students’ social–emotional competencies [46,47,54], this study aimed to investigate whether it could also lead to gains in students’ social–emotional competencies and to a reduction in internalizing and externalizing problems in the context of the COVID-19 pandemic. Although the initial design of the study included a control group, it had to be changed for ethical reasons, as there was strong pressure from schools for all students to benefit from the program during the pandemic period.

While conducting the study, we also examined whether children (boys and girls) with varying levels of social–emotional competencies and externalizing and internalizing problems and from different socioeconomic backgrounds were differently affected by the program. The following research questions were therefore formulated: (a) Do students with lower baseline levels of social–emotional competencies and higher baseline levels of externalizing and internalizing problems profit better? (b) Do boys reveal more gains than girls? (c) Do students from low-SES backgrounds display more gains than students from high-SES backgrounds?

## 2. Materials and Methods

### 2.1. Participants

Three hundred and fifty-eight [184 boys (51.4%)] third- and fourth-grade students (*M*_age_ = 8.56; *SD* = 0.82) from two Portuguese primary state school clusters in the central and southern regions of the country with five and three elementary schools, respectively, participated in this study (17 classes). The total number of students per school ranged from 18 to 67. SES was somewhat heterogeneous, but predominantly middle class [21.6% (*n* = 75) low SES; 31% (*n* = 108) medium SES; and 47.4% (*n* = 165) high SES]. Schools varied slightly in ethnicity (most students were Caucasian, but there were also black/African Portuguese students, and some Eastern European, Asian, and Roma minorities). Overall, 65% (*n* = 195) of the fathers and 76% (*n* = 242) of the mothers of these students had completed high school and/or obtained higher education.

### 2.2. Procedure

#### 2.2.1. Implementation Procedures

The “Slowly but Steadily” program was adopted by the Gulbenkian Knowledge Academies as a benchmark methodology for promoting social–emotional competencies in elementary school children based on evidence of its effectiveness, as shown in previous studies [46,47]. The Calouste Gulbenkian Foundation launched the challenge of supporting the Gulbenkian Knowledge Academies throughout the country with the aim of promoting artistic, scientific, community, cultural, and sports activities in areas as diverse as education and health, involving social or technological issues that develop competencies such as critical thinking, communication, resilience, teamwork, overcoming frustration, and mastering the ability to solve complex problems or adapt to change in children and young people under the age of 25 years. The support provided to all the Academies was technical, financial, and of a mentoring nature. Accordingly, a competition was opened to 100 Gulbenkian Knowledge Academies over three years, and in one of those years, four Academies were selected to implement the “Slowly but Steadily” program in the school context. The data reported herein refer to two of these Academies, which signed a collaboration protocol with the Calouste Gulbenkian Foundation for this purpose. These two Academies were two school clusters in the central and southern regions of the country, with five and three elementary schools, respectively.

The program facilitators received 16 h of initial training that included information on the theoretical rationale of the science of prevention and SEL, experiential activities to promote social and emotional competencies, methods to conduct reflection on activities of this nature, and the program, its history, objectives, the results obtained in terms of scientific evidence, and its implementation. In addition to the initial training, continued technical support was provided in the form of monthly supervision in groups of two/three facilitators.

The program was implemented during school hours in the presence of the respective teachers as part of the curriculum. The weekly sessions were supported by two school psychologists with experience in group intervention with third- and fourth-grade children, with the help of three junior psychologists.

#### 2.2.2. Data Collection Procedures

Ethical standards were ensured by the Calouste Gulbenkian Foundation committee, and the project was approved by the Ethics and Deontology Committee of the Faculty of Psychology, University of Lisbon. Letters were sent to the children’s homes to inform parents/guardians of the nature and purpose of the study. All eight schools utilized passive informed consent since the program had already been accepted as part of the school curriculum, in line with national legislation. Verbal assent was obtained from the children, and no incentives for participation were offered.

Data collection was planned to occur according to a 2 (interventions vs. control) × 2 (pre- vs. post-test) quasi-experimental design, as sampling was not totally random and not all school/class effects could be controlled. Both groups were tested at baseline under the same conditions. Unfortunately, due to the COVID-19 pandemic and first lockdown, the intervention was interrupted in that school year, and a new research design was established in the following school year with other students. For ethical and social responsibility purposes, all the participants involved in the study had access to the intervention. Hence, the present study has a level 6 quality of evidence for proof of efficacy as a result of the absence of a control group [55].

Multi-method, multi-agent assessments were gathered at baseline and post-test points. The measures were administered by the facilitators to the children and teachers during the second (following an initial presentation session) and last sessions of the program, with a pre–post period of 8 months and a 2 month interruption, roughly in the middle of the program’s implementation, due to the second lockdown. During the completion of the questionnaire, the instructions were read aloud to the children to lessen the effects of their reading skills on their understanding of the items. The teachers completed three questionnaires regarding each of their students. Demographic data were collected at the pre-test point.

### 2.3. Measures

The data were collected through self-report (students) and hetero-report (teachers) questionnaires before and after the program’s implementation to evaluate social–emotional competencies (emotional knowledge and social competence) and psychological adjustment (anxiety, aggressiveness, and social problems). Program satisfaction was also measured. All the measures had been used in previous studies with Portuguese participants, revealing acceptable to good reliability and validity. Higher scores reflected higher levels of social–emotional competencies and program satisfaction and lower levels of psychological adjustment. Socio-demographic data were also collected at the pre-test point (gender, age, school year, parents, schooling, and profession).

#### 2.3.1. Emotional Knowledge

Emotional knowledge was assessed through the Assessment of Children’s Emotions Scales (ACES [56]; Portuguese adaptation [57]). This measure evaluated children’s emotion perception accuracy (EPA) and included subscales concerning social behaviors (15 items; e.g., Jeff is being nice to everybody), social situations (15 items; e.g., Mary’s grandfather died), and facial expressions (20 items/photographs in the Portuguese adaptation). In response to each item, children labeled the protagonist’s feeling by selecting from happy, sad, mad, scared, or no feeling options. This is a measure of maximal behavior since it requires respondents to complete a task that taps into emotional knowledge. The overall EPA score reflects how often a child answered the 40 items (the “no feeling” items were not included in the EPA score) correctly for joy, sadness, anger, and fear across the three sections (*KR*-20 pre-test = 0.94, *KR*-20 post-test = 0.95). For ACES, reliability was computed through the Kuder–Richardson 20 coefficient, as it is equivalent to Cronbach’s alpha but adequate for dichotomic data [58].

#### 2.3.2. Social Competence

Social competence was evaluated by teachers through scale A of the School Social Behavior Scales (SSBS-2 [59]; Portuguese adaptation [54]). The SSBS-2 is composed of 32 items (Cronbach’s αpre-test = 0.98, αpost-test = 0.98) organized into three subscales and describes adaptive or positive behaviors that are likely to lead to positive personal and social outcomes. The abilities of self-control, self-restraint, cooperation, and compliance with the demands of school rules and expectations were assessed through the self-management/compliance subscale (SM; 10 items; Cronbach’s αpre-test = 0.96, αpost-test = 0.96). Social competence needed to establish positive relationships with and gain social acceptance from peers (e.g., interacts with a wide variety of peers) was measured through the peer relations subscale (PR; 14 items; Cronbach’s αpre-test = 0.96, αpost-test = 0.96). Lastly, the ability to engage in academic tasks (e.g., completes school assignments on time) and be academically competent was assessed through the academic behavior subscale (AB; 8 items; Cronbach’s αpre-test = 0.95, αpost-test = 0.94). Items were rated using a 5-point scale (1 = never to 5 = frequently).

#### 2.3.3. Anxiety

The State-Trait Anxiety Inventory for Children (STAI-C [60]; Portuguese adaptation [61]) assessed the intensity of children’s trait anxiety cognitions and symptoms by using only the second half of the full STAI-C measure (20 items; Cronbach’s αpre-test = 0.79, αpost-test = 0.80). Items (e.g., it is difficult for me to face my problems) were rated by students using a 3-point scale (1 = very little of the time to 3 = a lot of the time).

#### 2.3.4. Aggressiveness

Children’ aggressiveness was evaluated by teachers through a six-item scale (Aggressive Behaviors Questionnaire [62]) that assessed the frequency of direct and indirect forms of aggressive behaviors (Cronbach’s αpre-test = 0.94, αpost-test = 0.93). Items (e.g., provokes or threats peers) were rated using a 5-point scale (1 = never to 5 = frequently).

#### 2.3.5. Social Problems

The 10-item social problems subscale of the Teachers Report Form (TRF [63]; Portuguese adaptation [64]) was completed by teachers to assess students’ social, behavioral, and emotional externalizing problems (e.g., does not get along with other kids) on a 3-point rating scale (0 = not true to 2 = frequently true), (Cronbach’s αpre-test = 0.98, αpost-test = 0.98).

#### 2.3.6. Program Satisfaction

Program satisfaction was evaluated through a three-item (e.g., Did you like the Slowly but Steadily program?) self-report questionnaire (Cronbach’s α = 0.63) for students. Responses to the items were measured on a 5-point scale (1 = not at all to 5 = very much).

### 2.4. Data Analysis

Analysis of the Q-Q plots provides evidence that the data presented a tendency toward a normal distribution (i.e., |*z*| < 3) [65] and were not impacted by outliers, thus allowing for the use of parametric statistical analysis. Regarding missing data, the Little’s MCAR test revealed that missing data were not distributed completely at random [*χ*^2^ (41,718) = 43,559.473, *p* < 0.001]. Additionally, for some items, the percentage of missing data was above 5%. This suggests that the missingness is related to the observed data. Thus, to handle missing data, the expectation–maximization (EM) technique was used to estimate and input missing values, since it is a widely used and powerful technique in statistics [66]. A one-way ANOVA was conducted to test baseline differences across gender and SES. Effect sizes were calculated as partial eta-squared (*η*_p_^2^) and were interpreted as small, medium, and large effects, respectively, for values of 0.01, 0.06, and 0.14. To test for the interaction effect of the baseline level of the assessed variables, participants were categorized into 4 levels defined based on the pre-test quartiles (level 1, below 1st quartile; level 2, between 1st and 2nd quartiles; level 3, between 2nd and 3rd quartiles; and level 4, above 3rd quartile). Data analyses were conducted using linear mixed-effects models with lme4 [67] and lmerTest [68] packages in R-Studio (Version 4.4.1) [69]. Participants were included as random effects, whereas time, gender, baseline level of the assessed variables, and SES level were included as fixed effects. Confidence intervals were computed using the “boot” R package [70]. A follow-up simple slope analysis was conducted to explore significant interactions. No part of the study analysis was pre-registered prior to the research being conducted.

## 3. Results

No differences were found between gender [*F*(1, 342) = 0.181, *p* = 0.671] and SES [*F*(2, 342) = 0.336, *p* = 0.715] in terms of program satisfaction (*M* = 4.68, *SD* = 0.35), with results suggesting strong acceptance on the part of the students.

### 3.1. Baseline Differences across Gender and SES

Baseline differences were found between girls and boys for anxiety, aggressiveness, social competence, peer relations, self-management, and academic behavior, where the girls presented higher means than the boys in all the variables except for aggressiveness (Table 1).

Regarding SES, differences were found for emotional knowledge, aggressiveness, social problems, social competence, peer relations, self-management/compliance, and academic behavior (Table 2). Post-hoc comparisons showed that for all the variables, high SES differed from low SES (but not from medium SES) in favor of students with a high SES. Moreover, regarding social problems and academic behavior, medium SES also differed from low SES in favor of students with a medium SES. No differences were found between students with a high and medium SES.

### 3.2. Pre–Post-Test Gains

Table 3 depicts means and standard deviations of the outcome variables.

#### 3.2.1. Emotional Knowledge

**Figure 1:**
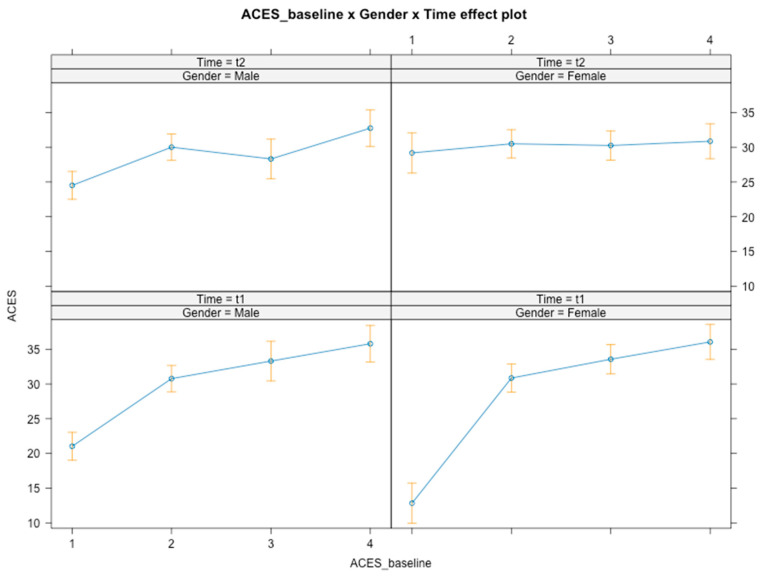
Graphical representation of interaction effects (time × gender × baseline level) on emotional knowledge.

Time × gender × baseline level interactions were found for emotional knowledge [*F*(3, 348) = 7.714, *p* < 0.001] (Figure 1). Overtime, participants with low baseline emotional knowledge improved at the post-test point, but top-tier participants showed a decrease in emotional knowledge after the intervention (*B* = −11.48, *SE* = 5.60, *t*(348) = −2.051, *p* = 0.041, 95% CI [−23.33, −0.36]). Girls in the top-tier level of emotional knowledge (*B* = 30.85) showed a greater decrease in emotional knowledge at the post-test point in comparison to boys (*B* = 32.74) and lower-competence participants. That said, there was a significant global increase in emotional knowledge overtime, but this was only found to be true for participants with baseline low emotional knowledge. No interaction effects were found for SES level.

#### 3.2.2. Social Competence

**Figure 2:**
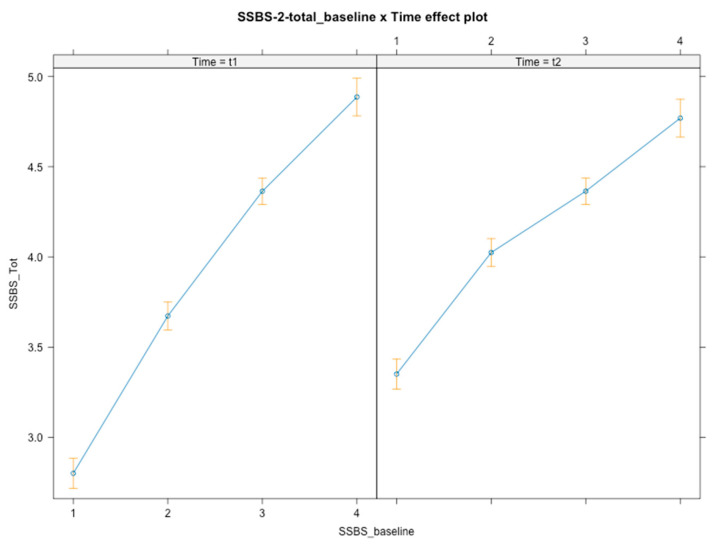
Graphical representation of interaction effects (time × baseline level) on social competence.

Time × baseline level interactions were found [*F*(3, 348) = 28.33, *p* < 0.001], with students at a lower baseline level increasing the most after the intervention (*B*_t1_ = 2.80, *B*_t2_ = 3.35) compared to students with a higher baseline level of social competence (Figure 2). These students, despite maintaining global higher scores of social competence, tended to maintain their social competence (3rd tier: *B* = −0.43, *SE* = 0.13, *t*(348) = −3.386, *p* < 0.001, 95% CI [−0.68, −0.18]; *B*_t1_ = 4.36, *B*_t2_ = 4.36) or exhibit a slight decrease (4th tier: *B* = −0.56, *SE* = 0.13, *t*(348) = −4.355, *p* < 0.001, 95% CI [−0.84, −0.30]; *B*_t1_ = 4.88, *B*_t2_ = 4.77) following the intervention. No interaction effects were found for gender or SES.

#### 3.2.3. Peer Relations

**Figure 3:**
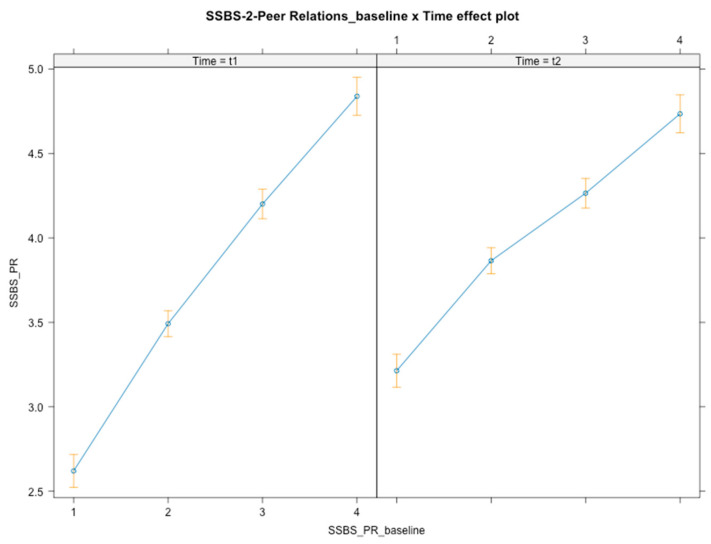
Graphical representation of interaction effects (time × baseline level) on peer relations.

Time × baseline level interactions were found [*F*(3, 348) = 20.65, *p* < 0.001] (Figure 3), with students at a lower baseline level increasing the most following the intervention (*B*_t1_ = 2.62, *B*_t2_ = 3.21) compared with top-tier level students (*B* = −0.56, *SE* = 0.14, *t*(348) = −3.870, *p* < 0.001, 95% CI [−0.83, −0.28]), whose competence slightly decreased after the intervention (*B*_t1_ = 4.84, *B*_t2_ = 4.73). Overall and over time, students with lower baseline competencies were those who profited more from the intervention; in contrast, the higher the baseline level, the lower the post-intervention gain. No interaction effects were found for gender or SES.

#### 3.2.4. Self-Management/Compliance

**Figure 4:**
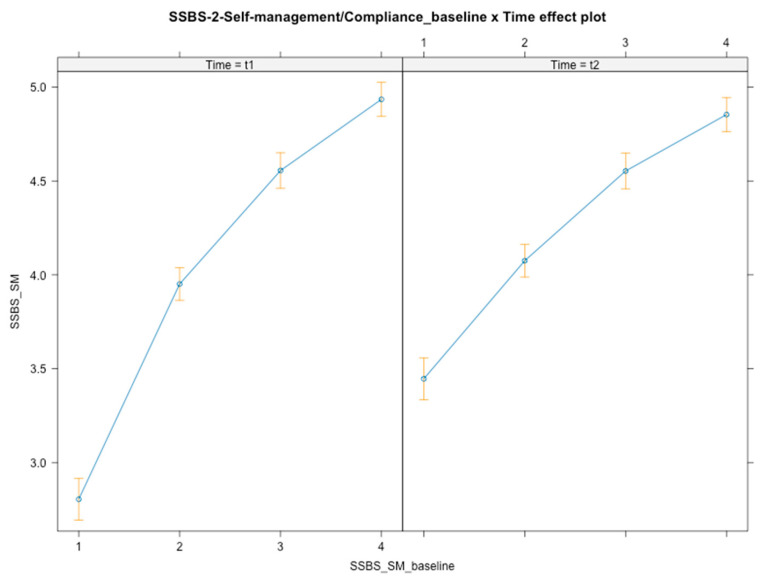
Graphical representation of interaction effects (time × baseline level) on self-management/compliance.

Time × baseline level interactions were found [*F*(3, 348) = 27.02, *p* < 0.001] (Figure 4), with students at a lower baseline level increasing the most following the intervention (*B*_t1_ = 2.80, *B*_t2_ = 3.45) compared with all 2nd-tier (*B* = −0.56, *SE* = 0.13, *t*(348) = −4.178, *p* < 0.001, 95% CI [−0.81, −0.28]; *B*_t1_ = 3.95, *B*_t2_ = 4.07), 3rd-tier (*B* = −0.51, *SE* = 0.13, *t*(348) = −3.809, *p* < 0.001, 95% CI [−0.79, −0.24]; *B*_t1_ = 4.56, *B*_t2_ = 4.55), and 4th-tier (*B* = −0.62, *SE* = 0.13, *t*(348) = −4.641, *p* < 0.001, 95% CI [−0.87, −0.29]; *B*_t1_ = 4.94, *B*_t2_ = 4.85) students. Overall and over time, students with lower baseline self-management/compliance competencies were those who profited more from the intervention, in contrast to those at the top-tier baseline levels who faced a slightly decreased competence following the intervention. No interaction effects were found for gender or SES.

#### 3.2.5. Academic Behavior

**Figure 5:**
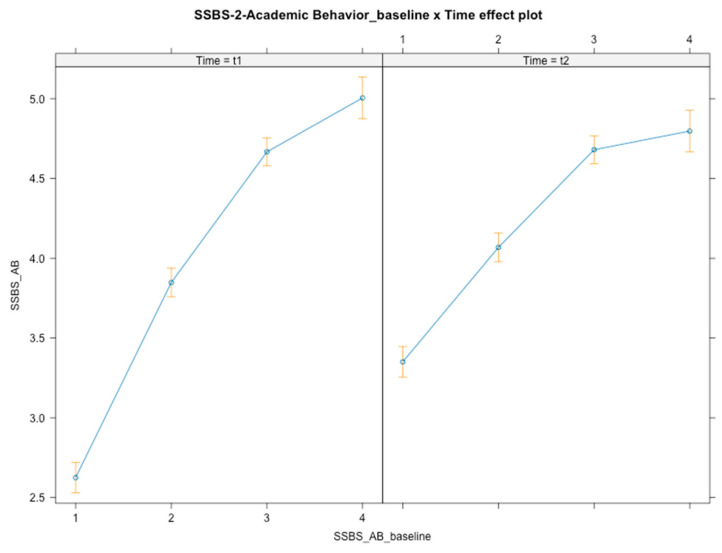
Graphical representation of interaction effects (time × baseline level) on academic behavior.

Time × baseline level interactions were found [*F*(3, 348) = 34.19, *p* < 0.001] (Figure 5), with students at a lower baseline level increasing the most following the intervention (*b*_t1_ = 2.63, *b*_t2_ = 3.35) compared with all 2nd-tier (*B* = −0.52, *SE* = 0.14, *t*(348) = −3.680, *p* < 0.001, 95% CI [−0.82, −0.26]; *B*_t1_ = 3.84, *B*_t2_ = 4.07), 3rd-tier (*B* = −0.76, *SE* = 0.15, *t*(348) = −5.057, *p* < 0.001, 95% CI [−1.08, −0.46]; *B*_t1_ = 4.67, *B*_t2_ = 4.68), and 4th-tier (*B* = −0.94, *SE* = 0.16, *t*(348) = −6.121, *p* < 0.001, 95% CI [−1.25, −0.67]; *B*_t1_ = 5.01, *B*_t2_ = 4.80) students. Overall and over time, students with lower baseline levels of academic behavior were those who profited more from the intervention, in contrast to those at the top-tier baseline level, who faced a slightly decreased competence following the intervention. No interaction effects were found for gender or SES.

#### 3.2.6. Anxiety

**Figure 6:**
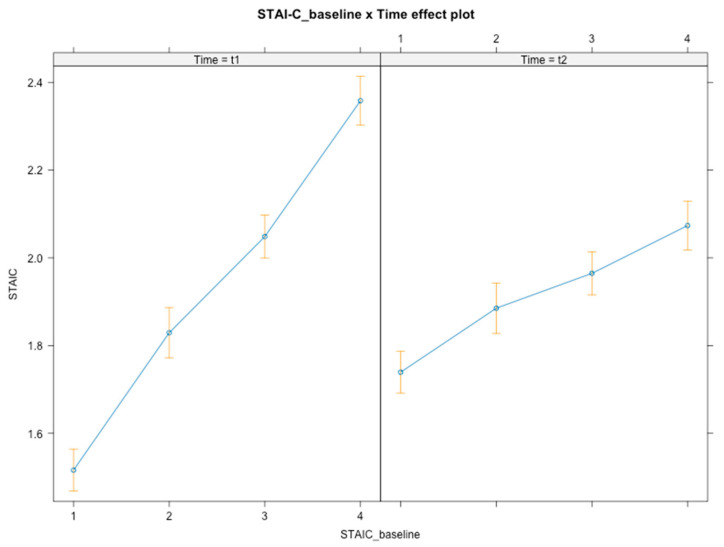
Graphical representation of interaction effects (time × baseline level) on anxiety.

Time × baseline level interactions were found [*F*(3, 348) = 37.99, *p* < 0.001] (Figure 6), with students from both levels 3 (*B* = −0.33, *SE* = 0.09, *t*(348) = −3.681, *p* < 0.001, 95% CI [−0.49, −0.15]) and 4 (*B* = −0.33, *SE* = 0.10, *t*(348) = −3.217, *p* = 0.001, 95% CI [−0.53, −0.13]) revealing a decrease in anxiety compared to level-1 students, who slightly increased their anxiety level following the intervention. Globally, over time, students who had lower baseline levels of anxiety increased their anxiety level (although remaining at an overall lower level; *B*_t1_ = 1.52, *B*_t2_ = 1.74), while students with higher baseline levels of anxiety decreased their anxiety levels following the intervention (3^rd^ tier: *B*_t1_ = 2.05, *B*_t2_ = 1.96; 4^th^ tier: *B*_t1_ = 2.36, *B*_t2_ = 2.07). No interaction effects were found for gender or SES.

#### 3.2.7. Aggressiveness

No effects of time were found on aggressiveness (*B* = −0.06, *SE* = 0.08, *t*(348) = 0.786, *p* = 0.432, 95% CI [−8.57, 0.23]).

#### 3.2.8. Social Problems

**Figure 7:**
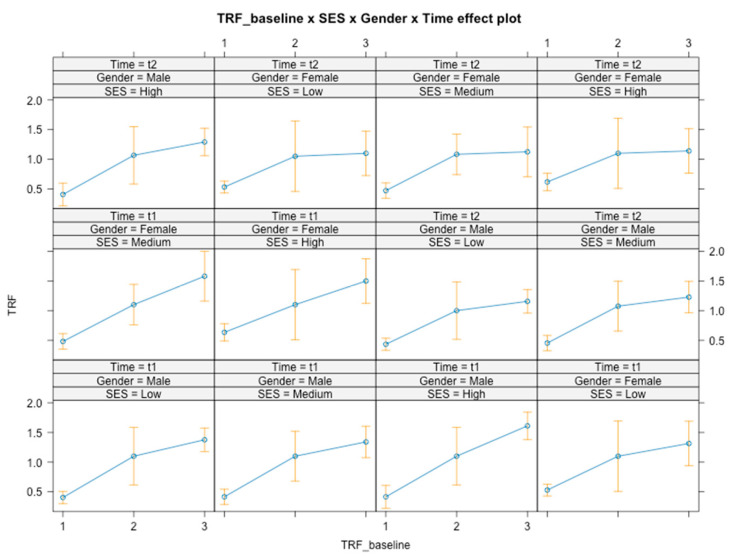
Graphical representation of interaction effects (time × gender × SES × baseline level) on social problems.

Time × gender × SES × baseline level interactions were found [*F*(4, 348) = 2.36, *p* = 0.05] (Figure 7). Following the intervention, there was an overall decrease in social problems (*B* = −0.07, *SE* = 0.10, *t*(348) = −2.965, *p* = 0.003, 95% CI [−0.55, −0.12]), mainly for girls with medium SES who had a higher baseline level (*B*_t1_ = 1.58, *B*_t2_ = 1.12).

## 4. Discussion

The findings provide considerable support for the “Slowly but Steadily” program, highlighting significant intervention gains. First, the average student who participated in this SEL program improved, as expected, in some social and emotional competencies (such as social competence, peer relations, self-management, academic behavior) and externalizing problems (social problems). No general post-intervention improvement was observed in emotional knowledge, anxiety, or aggressiveness, and no unexpected negative effects were identified.

Our results are in line with the findings reported in previous studies regarding the efficacy of the “Slowly but Steadily” program, showing the improvement of some social and emotional competencies and the reduction in externalizing problems, with small to moderate effect sizes [46,47]. Taken together, these findings are also consistent with studies from other countries, partially supporting the cross-cultural generalization of SEL programs’ efficacy [13,29,30,31].

Similar results were also yielded by other studies on universal approaches in schools, which had fewer effects on anxiety [71] than targeted interventions [34,72]. Moreover, given that clinically significant anxiety rates doubled during the pandemic, with one in five young people experiencing anxiety and with evidence of higher rates in children [4], this may also mean that the severity of symptoms makes it even more challenging for universal programs to positively impact this internalizing problem.

Another important research goal was to ascertain whether the intervention is more effective for some students than for others. Accordingly, second, students with lower social–emotional competencies and psychological adjustment were found to profit more from the program in all the variables studied except for aggressiveness. These findings are consistent with those of other studies regarding the moderating effect of baseline levels of self-reported symptoms of anxiety [73]. However, following prior literature, effects on aggressiveness were also expected [74]. Our results are also in line with evidence collected during the pandemic crisis showing that the children and adolescents who faced higher risks of psychological health problems were those with previous psychological health problems [4]. Furthermore, although students with above-average social–emotional competencies and below-average externalizing and internalizing problems at the pre-test point did not improve these results directly through the program, they may also have benefited indirectly, as they now share their environment with classmates who have enhanced some of their social–emotional competencies and reduced some of their previous externalizing problems as a result of the program [75].

Third, the data show that gender moderated the effect of the program on emotional knowledge, with girls with lower baseline levels improving more than boys, and on social problems, with girls with medium SES retreating more than boys. No significant gender effect was found favoring boys in self-management, aggressiveness, or social problems, contrary to the findings of a previous study on the “Slowly but Steadily” program’s efficacy [46]. However, some caution should be taken when directly comparing these two studies, since a control group was used in the previous study. In a review article on social–emotional competence [76] focusing on what is known about effective intervention approaches, the research data (assessed in three of the five studies) reflected that intervention effects were comparable for students of both genders.

Fourth, this study’s results corroborate the premise that “high-SES” children profit as much as “low-SES” children from the program, except with regard to social problems, in keeping with previous studies on this program [46,47]. Furthermore, the findings also support two meta-analysis studies and a review article suggesting that school-based social–emotional competence interventions are suitable and effective for all students [29]; that positive effects are found across all demographic subgroups, including SES status [31]; and that the intervention effects are comparable for students of different ethnicities [76].

“Slowly but Steadily” was largely developed based on the primary components of SEL programs and followed CASEL (Collaborative Academic Social and Emotional Learning) recommendations, especially regarding SAFE practices [29] and the careful monitoring of program delivery. This program is already backed by previous studies showing its efficacy in promoting social and emotional competencies and effectiveness in reducing externalizing behaviors and improving academic performance; however, this is the first study to analyze its effects during the pandemic period. One strength of the present study was the 16 h initial training of the facilitators and teachers, as well as the ongoing monthly supervision and technical support in groups of two/three facilitators. One of the factors that may have a very significant impact on how SEL programs are implemented is the presence of a consultant/supervisor. This professional can be a valuable asset in helping to build the capacity of the school team to function within a culture of integrity by ensuring regular meetings with the participation of all members and by examining the indicators of effective practices. He or she can also help the team understand those indicators, develop tasks that lead to full implementation, and report evidence of that implementation in a suitable manner [77].

Furthermore, “Slowly but Steadily” has social validity since it has been highly accepted by students and regarded as a meaningful program that meets their needs, regardless of their gender or SES. Another strength of the study was the use of multiple informants (the participants themselves and their teachers) and methods (self-reports, knowledge assessment, and behavior ratings), which contribute to reducing common method and source biases [41,78]. This is especially notable bearing in mind that in the most recent large-scale meta-analyses of SEL programs [31], almost three-quarters of the studies (i.e., 72.2%) relied on self-report measures to evaluate student outcomes. Particularly noteworthy was the use of a measure of maximal behavior, which covered the emotional knowledge construct in order to reduce bias and social desirability [35]. The teachers who collected the ratings did not deliver the intervention, which lends credibility to the findings, since it reduces the probability of an expectancy effect [79], which poses a threat to internal validity [80].

One limitation of the present study is the absence of a control group. The initial design of the study included a control group, but this had to be changed for ethical reasons, since the program was implemented during the pandemic period. Additionally, due to the high risks to children’s socioemotional development and mental health/well-being, the program’s implementation sought to target the maximum number of students. Another limitation is that the program was implemented over the course of one single academic year. Moreover, more involved attempts should be made in the future to promote greater generalization by means of daily activities carried out by teachers. Future studies should prioritize a multi-year intervention program for all elementary school years involving a whole-school approach, the inclusion of parental reports (triangulated assessment), and a control group. The duration of gains should also be analyzed through follow-up assessments to ascertain whether the effects remain statistically significant and if there are any potential “sleeper effects”. Although it was ensured that all teachers involved in the study had the same training as the program implementers, to facilitate the sustainability of the program after the project’s funding ended, their degree of involvement throughout the sessions varied greatly from teacher to teacher.

Following the recommendations in prior literature (e.g., [38]), the explicit training of teachers for SEL is of great relevance, and this study bridges this gap by including 16 h of initial training for facilitators and teachers. In fact, SEL is still lacking in both the initial and continuing training of teachers, with teachers frequently feeling unprepared to respond to their students’ social and emotional needs (e.g., [38]), thus contributing to teacher burnout [81]. Bridging this gap is essential, since recent literature points to both the direct impacts of SEL on teachers’ personal (e.g., well-being [82]) and professional (e.g., occupational health [81]) outcomes and the indirect impacts on their students’ social–emotional competencies, well-being, and academic performance (e.g., [83,84]).

Additionally, since the “Slowly but Steadily” program received funding from the Gulbenkian Knowledge Academies, it was possible to hire the services of an external consultant with over 15 years of professional experience in the implementation of SEL programs in schools, who supervised the implementation of the program monthly in small groups of two to three supervised professionals. The literature highlights that external consultants should be selected according to their experience in implementing programs of this nature in school settings [85], helping educational agents learn the active principles of the theory behind the intervention, properly implementing a program in different situations, identifying when parts of it need to be repeated, and understanding what kind of modifications are acceptable, and perhaps even necessary, to adjust the intervention to particular contexts [86]. The difficulties encountered by some school institutions are most likely the same as those overcome in others, and consultants may serve as mentors to the former [85], as was the case in this implementation of the “Slowly but Steadily” program.

Finally, there is a gap between research and practice regarding the prevention and promotion of skills in school settings [87]. In Portugal and the rest of Europe, this phenomenon stems from the low level of coordination between researchers and professionals, the absence of a culture of publication of results by professionals, and the scarce use of methodologies to assess the efficacy and effectiveness of programs and the consequent dissemination of the results generated by their implementation [88]. This study is part of a substantial financial effort on the part of a private institution of public utility, the Calouste Gulbenkian Foundation, for the promotion of social and emotional skills in children and young people under the age of 25 years throughout the country. This effort was not only employed within the more common realm of funding the studies of research centers aimed at designing, implementing, and evaluating the efficacy of programs of this nature in educational institutions. In this case, the funding was directly allocated to the institutions where the children and young people spend most of their time (mostly, but not exclusively, schools), following their selection within the scope of a call for applications. The funding covered not only the direct costs of applying the program but also the initial training, monthly technical supervision, and continuous supervision, monitoring, and evaluation with a research team set up for this purpose, as well as the publication and dissemination of the results, thus highlighting the importance of this major national contribution.

## 5. Conclusions

The "Slowly but Steadily" program received considerable support from our findings, which suggested that the delivery of this universal SEL program over one academic year partially improves the social–emotional competencies and psychological adjustment of third- and fourth-grade children, in the context of the COVID-19 pandemic. The results also reveal that a child’s distance from acquiring appropriate social–emotional competencies and being psychologically well adjusted (baseline levels) was a strong moderator of all the variables analyzed, while gender and SES only moderated emotional knowledge and social problems.

Managing our emotions and relating to others are among the greatest challenges we face in life. The identification of universal programs with promising quality and efficacy is crucial nowadays, as well as guiding schools toward making informed choices regarding the adoption, implementation, and evaluation of SEL programs [89]. Ideally, schools should focus on evidence supporting these programs in the contexts in which they are efficient [35,90].

## Figures and Tables

**Table 1:** Means and standard deviations of the outcome variables and one-way ANOVAs for pre-test mean differences by gender.

Outcomes	Gender	*M* (*SD*)		*F*	*p*	*η_p_* ^2^
		Pre-Test	Post-Test			
Emotional Knowledge	Girl	29.25 (9.87)	30.48 (8.70)	0.036	0.850	0.003
	Boy	29.42 (7.17)	28.92 (9.95)			
Social Competence	Girl	4.13 (0.73)	4.26 (0.65)	25.559	<0.001	0.07
	Boy	3.70 (0.86)	3.95 (0.77)			
Peer Relations	Girl	4.01 (0.77)	4.16 (0.70)	23.799	<0.001	0.06
	Boy	3.58 (0.87)	3.86 (0.81)			
Self-Management/ Compliance	Girl	4.30 (0.69)	4.40 (0.65)	37.154	<0.001	0.10
	Boy	3.76 (0.96)	3.97 (0.86)			
Academic Behavior	Girl	4.12 (0.92)	4.26 (0.76)	8.398	0.004	0.02
	Boy	3.83 (0.96)	4.07 (0.84)			
Anxiety	Girl	1.97 (0.35)	1.91 (0.35)	5.719	0.017	0.02
	Boy	1.88 (0.33)	1.91 (0.34)			
Aggressiveness	Girl	1.22 (0.47)	1.14 (0.35)	42.909	<0.001	0.11
	Boy	1.70 (0.84)	1.46 (0.63)			
Social Problems	Girl	0.64 (0.53)	0.61 (0.50)	0.520	0.471	0.00
	Boy	0.68 (0.60)	0.65 (0.53)			

**Table 2:** Means and standard deviations of the outcome variables and one-way ANOVAs for pre-test mean differences by SES.

Outcomes	SES	*M* (*SD*)		*F*	*p*	*η_p_^2^*
		Pre-Test	Post-Test			
Emotional Knowledge	High	30.52 (8.05)	31.32 (7.29)	3.728	0.025	0.02
	Medium	28.73 (8.71)	29.79 (9.09)			
	Low	27.40 (9.57)	25.53 (12.72)			
Social Competence	High	4.07 (0.77)	4.25 (0.69)	7.389	<0.001	0.04
	Medium	3.92 (0.84)	4.11 (0.76)			
	Low	3.63 (0.85)	3.84 (0.65)			
Peer Relations	High	3.94 (0.84)	4.14 (0.75)	5.843	0.003	0.03
	Medium	3.79 (0.85)	3.99 (0.85)			
	Low	3.54 (0.83)	3.80 (0.63)			
Self-Management/ Compliance	High	4.14 (0.82)	4.30 (0.77)	3.669	0.026	0.02
	Medium	4.04 (0.88)	4.21 (0.81)			
	Low	3.82 (0.95)	3.96 (0.77)			
Academic Behavior	High	4.20 (0.86)	4.38 (0.71)	11.857	<0.001	0.06
	Medium	3.98 (0.98)	4.20 (0.83)			
	Low	3.57 (0.98)	3.76 (0.80)			
Anxiety	High	1.89 (0.34)	1.91 (0.36)	1.577	0.208	0.01
	Medium	1.92 (0.33)	1.92 (0.32)			
	Low	1.97 (0.37)	1.90 (0.34)			
Aggressiveness	High	1.38 (0.64)	1.22 (0.47)	4.282	0.015	0.02
	Medium	1.44 (0.69)	1.36 (0.60)			
	Low	1.67 (0.89)	1.38 (0.56)			
Social Problems	High	0.61 (0.55)	0.59 (0.51)	4.386	0.013	0.03
	Medium	0.63 (0.55)	0.61 (0.51)			
	Low	0.84 (0.60)	0.75 (0.51)			

**Table 3:** Means and standard deviations of the outcome variables.

Outcomes	*M* (*SD*)	
	Pre-Test	Post-Test
Emotional Knowledge	29.34 (8.58)	29.68 (9.39)
Social Competence	3.91 (0.82)	4.10 (0.73)
Peer relations	3.79 (0.85)	4.01 (0.77)
Self-Management/Compliance	4.03 (0.88)	4.18 (0.79)
Academic Behavior	3.97 (0.95)	4.16 (0.81)
Anxiety	1.92 (0.35)	1.91 (0.34)
Aggressiveness	1.47 (0.72)	1.30 (0.54)
Social Problems	0.66 (0.57)	0.63 (0.51)

## Data Availability

The raw data that support the findings of this study are available from the corresponding author upon reasonable request.
